# Nosocomial Bacteria Inhibition with Polymyxin B: In Silico Gene Mining and In Vitro Analysis

**DOI:** 10.3390/antibiotics13080745

**Published:** 2024-08-08

**Authors:** Jayendra Chunduru, Nicholas LaRoe, Jeremy Garza, Abdul N. Hamood, Paul W. Paré

**Affiliations:** 1 Chemistry & Biochemistry Department, Texas Tech University, Lubbock, TX 79409, USA; 2 Department of Immunology & Molecular Microbiology, Texas Tech University Health Sciences Center, Lubbock, TX 79430, USAabdul.hamood@ttuhsc.edu (A.N.H.)

**Keywords:** multidrug-resistant bacteria, cationic non-ribosomal peptides, polymyxin B, biosynthesis

## Abstract

Multidrug-resistant bacteria present a significant public health challenge; such pathogens exhibit reduced susceptibility to conventional antibiotics, limiting current treatment options. Cationic non-ribosomal peptides (CNRPs) such as brevicidine and polymyxins have emerged as promising candidates to block Gram-negative bacteria. To investigate the capability of bacteria to biosynthesize CNRPs, and specifically polymyxins, over 11,000 bacterial genomes were mined in silico. *Paenibacillus polymyxa* was identified as having a robust biosynthetic capacity, based on multiple polymyxin gene clusters. *P. polymyxa* biosynthetic competence was confirmed by metabolite characterization via HPLC purification and MALDI TOF/TOF analysis. When grown in a selected medium, the metabolite yield was 4 mg/L with a 20-fold specific activity increase. Polymyxin B (PMB) was assayed with select nosocomial pathogens, including *Pseudomonas aeruginosa*, *Klebsiella pneumonia*, and *Acinetobacter baumaii*, which exhibited minimum inhibitory concentrations of 4, 1, and 1 µg/mL, respectively.

## 1. Introduction

Non-ribosomal peptides (NRPs), unlike traditional mRNA-ribosomal catalyzed peptides are uniquely assembled by large multi-modular non-ribosomal peptide synthetases (NRPSs) present in bacteria and filamentous fungi. A NRPS module contains several domains that catalyze adenylation, condensation, and a peptide-carrier protein, or thiolation. Initiation modules usually lack condensation domains, while termination modules often possess an extra thioesterase domain for cleaving the final product. Bacterial NRPSs are encoded by genes organized in an operon with NRPSs activating and incorporating a broad range of substrates, including standard and non-proteinogenic amino acids (d- and l-), fatty acids, α-hydroxy acids, α-keto acids, and heterocycles, contributing to the chemical and structural diversity of NRPs [1,2,3].

Genetic mining for non-ribosomal polypeptides involves the systematic survey of microbial genomes to identify biosynthetic gene clusters within NRPSs. Using bioinformatics software and computational tools (e.g., antiSMASH, PRISM, and ClusterFinder), genomic data were analyzed, and putative gene clusters involved in non-ribosomal polypeptide biosynthesis were identified. Antibiotics and secondary metabolite analysis shell (antiSMASH) recognizes open reading frames within input sequences based on sequence similarities, domain composition, and gene organization. Using diagnostic gene clusters and sequence domains, antiSMASH links the usage and incorporation of explicit amino acids with organism-specific NRPSs [4,5,6,7,8].

Cationic NRPs (CNRPs) maintain an overall positive charge, often containing both common and non-canonical positively charged amino acids (e.g., arginine, histidine, lysine, ornithine, and 2,4-diaminobutyric acid) [9,10]. Bacterially produced CNRPs often exhibit selective activity against Gram-negative bacteria while having low cross-resistance with existing antibiotics. Indeed, colistin, a CNRP from *Paenibacillus* spp., is a critical last-resort treatment against multidrug-resistant Gram-negative infections [11]. Additional CNRPs isolated from *Paenibacillus*, including laterocidine [12], tridecaptin [13], and paenibacterin [14], are currently in clinical trials.

While genome screening identifies species that are genetically competent to produce NRPs in silico, culture conditions stimulate or inhibit the biosynthesis of such secondary metabolites in vivo. In liquid culture, such bacterial chemical defenses are usually produced in the late log or early stationary phase. Media nutrient composition and concentration not only affect bacterial growth but also regulate the gene induction associated with NRPSs. An external source of amino acids, temperature settings, and light/atmospheric conditions can all modulate bacteria growth and NRP accumulation [15,16]. Some NRPSs contain genes that are activated by stress [17], chemicals [17], elicitors [18], genetic manipulations [19], and interactions with other microbes culturing conditions (co-culture) [20]. For example, polymyxin E from *Paenibacillus polymyxa* [21] is enhanced with exogenous starch. Such metabolites can be isolated from media by acid precipitation [22], use of resins [23,24,25], or surface extraction [26].

In this study, over 11,000 genomes were screened to identify genera that are genetically competent for the biosynthesis of PMB, a highly potent antibiotic. Liquid cultural conditions that allowed for the isolation of PMB were identified. The purified metabolite was then assayed against multidrug-resistant bacterial strains of medical significance.

## 2. Results

### 2.1. Genome Mining for Non-Ribosomal Polypeptides

**Figure 1:**
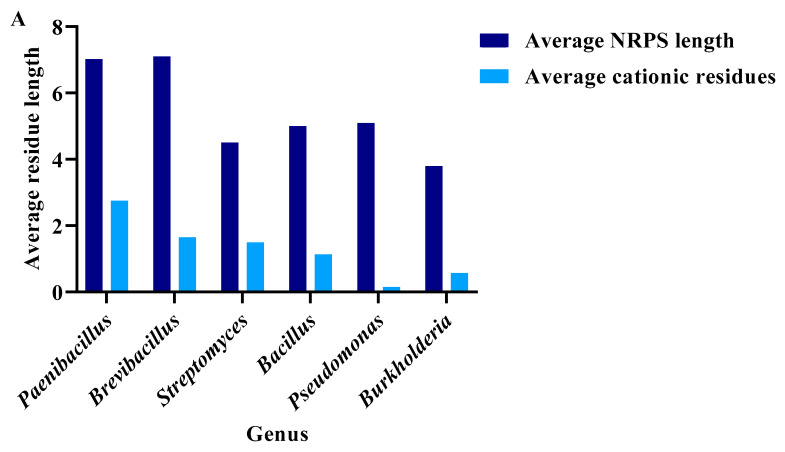

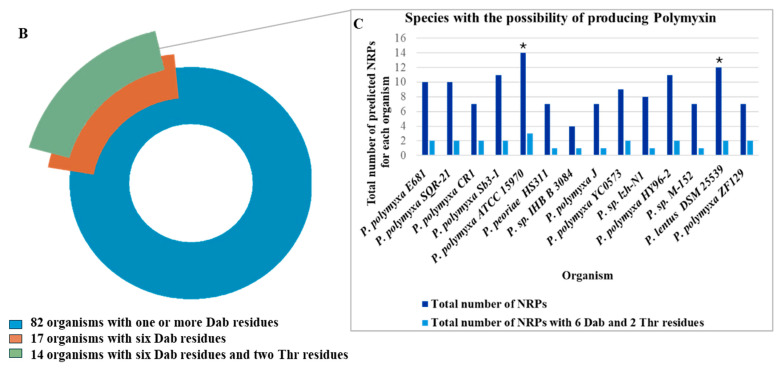
(**A**). Estimated non-ribosomal peptide length (average) in select genera (purple) and average cationic residues per peptide (blue): *Paenibacillus* n = 602 (range of peptide length = 1–30), *Brevibacillus* n = 249 (range of peptide length = 1–33), *Streptomyces* n = 2592 (range of peptide length = 1–56), *Bacillus* n = 4840 (range of peptide length = 1–26), *Pseudomonas* n = 1758 (range of peptide length = 1–48), and *Burkholderia* n = 1915 (range of peptide length = 1–28). (**B**). Fraction of bacteria that contain essential predicted residues for PPPB. legend for circle graph (**C**). Total number of organisms with the biological potential of producing polymyxin (* commercially available). Range of peptide length (n) (*P. lentus* DSM 25539 (1–15), *P.* sp. IHB B 3084, *P. polymyxa* CR1 (2–14), *P. polymyxa* ZF129 (3–13) *P. polymyxa* J (3–14), *P. polymyxa* Sb3-1 (4–12)*, P. polymyxa* SQR-21, ATCC 15970, HY96-2 (4–13), *P. polymyxa* E681, YC0136, *P. peoriae* HS311 (4–14), *P*. sp. lzh-N1 (4–16), and *P*. sp. M-152 (4–18).

Genome mining identified biosynthetic gene clusters (BGCs) that encoded for NRP synthetases. From over 11,000 genome sequences representing 209 genera, 6 genera showed an average of more than 0.5 cationic BGC per species. *Paenibacillus* contains an average of 10 non-ribosomal peptide (NRP) BGCs and 3 cationic BGCs per genus out of a relatively small sample size of 70 organisms, in which the complete genome sequence was available. In contrast, *Pseudomonas* was represented by 3140 organisms and had an average of one BGC per organism and less than one cationic BGC (Figure S1). In terms of NRPs, the average number of residues in *Paenibacillus*, *Brevibacillus*, *Streptomyces*, *Bacillus*, *Pseudomonas*, and *Burkholderia* was 7, 7, 4, 5, 5, and 4, respectively; the cationic residue average for each NRP was 3, 2, 1, 1, 0.1, and 0.5, respectively, highlighting variability in NRP length and cationic content across these microbial genera (Figure 1A).

### 2.2. Polymyxin B Biosynthetic Gene Clusters

The core structure of PMB contains 2,4-diaminobutyric acid (Dab). Eighty-two organisms were found to have at least one Dab in the predicted NRP structure. Of these, 17 species were predicted to have six Dabs, and 14 of the 17 species had at least two Thr residues, which are key to the polymyxin core (Figure 1B). Of the 14 organisms, *P. polymyxa* and *P. lentus* were commercially available. *P. polymyxa* exhibited 14 predicted non-ribosomal peptides (NRPs) and 3 polymyxin clusters (Table 1). Similarly, *P. lentus* exhibited 12 NRPs, accompanied by 2 polymyxin clusters. These data provided the rationale for selecting *P. polymyxa* for confirming metabolite accumulation and assaying for biological activity (Figure 1C).

### 2.3. Confirming PMB Competence

**Figure 2:**
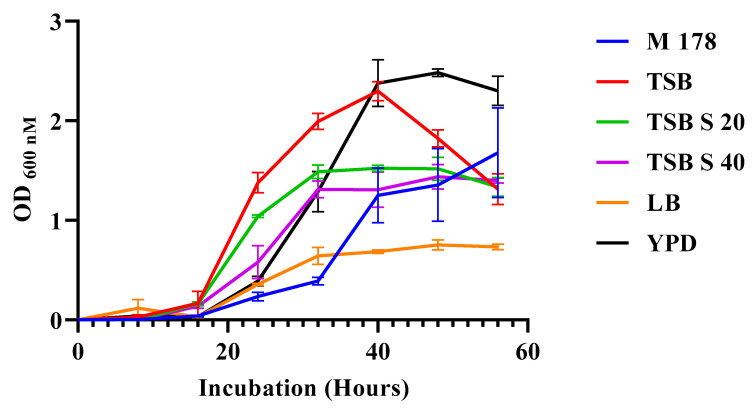
Media effect on *P. polymyxa* growth [ATCC-recommended media (M178, blue), tryptic soy broth (TSB, red), tryptic soy broth with starch (20 g/L) (TSB S20, green), tryptic soy broth with starch (40 g/L) (TSB S40, purple), Luria–Bertani broth (LB, orange), and yeast extract peptone dextrose (YPD, dark green)] (three trials in triplicate and error bars are SDs).

Bacterial growth and PMB accumulation were assayed with specific media supplements with an ATCC-recommended *Pseudomonas* media (M178) as a baseline substrate. The lag phase was invariant among the media tested (Figure 2). In the log phase, tryptic soy broth (TSB) and M 178 had the quickest and slowest cell accumulation, respectively. Yeast extract peptone dextrose (YPD) had a higher cell count after 56 h, while Luria–Bertani broth (LB) had the lowest count. Adding starch to TSB at 20 gm/L and 40 gm/L (TSB-S20 and TSB-S40, respectively) resulted in lower cell counts. The growth rate with starch was similar in both TSB-S20 and TSB-S40, where they reached the stationary phase at 36 h and stayed level until 56 h. Even though YPD had higher growth than the three TSB media, its total protein level was lower.

Protein release studies using the Bradford assay indicated that TSB yielded three times more protein than YPD and LB. Adding starch to TSB increased protein release, with a 2.2-fold enhancement for TSB with TSB-S20, while remaining constant for TSB with TSB-S40 after 56 h (Figure S2). Bioassay results confirmed that the addition of starch significantly boosted protein release, causing a 1.4-fold increase in specific activity for TSB-S20 and a 2-fold increase for TSB-S40 (Table 2). However, replicating the experiment with 40 g/L starch led to a problematic contamination, prompting the use of a concentration comprising 20 g/L starch.

### 2.4. NRP Antibiotic Isolation, Purification, and Characterization

**Figure 3:**
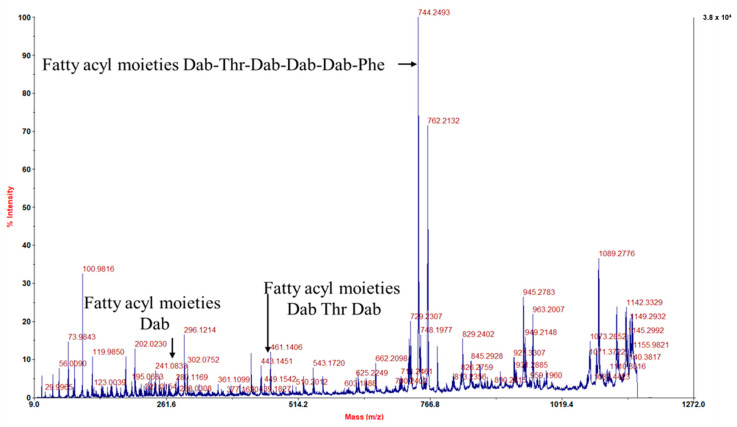
Fragmented MS/MS peaks using TOF of 1203.3698 Da peak; y-axis on right shows absolute intensity.

Acid precipitation from spent media had no antibiotic activity on *E. coli* nor the supernatant. The acidic extract from resins amberlite XAD-7, diaion HP-20, and MCI CHP-20 had antibiotic activity. Diaion HP-20 and MCI CHP-20 had similar antibiotic extraction, with their specific activity being 0.17 and 0.12 AU/mg, respectively. Amberlite XAD-7 resin had a 3.5-fold higher antibiotic extraction than the other two resins, with specific activity reported at 0.88 AU/mg (Table 2). HPLC fractions collected between 20 and 25 min fraction had an antibiotic activity (Figure S3), with a 10.5-fold increase regarding specific activity reported at 9.5 AU/mg. The isolated peptide exhibited antibiotic activity with a specific activity of 15.4 AU/mg and a 1.6-fold increase from the previous step. MALDI-TOF (time-of-flight) analysis revealed the presence of a compound with the following molecular weight of 1203.3698 Da (calculated for C_56_H_99_O_13_N_16_, [M + H]^+^, 1203.75720), (Figure S4), which matches PMB. Further confirmation of the structure was performed using TOF MS/MS analysis. Peak fragmentation provided structure identification with *m*/*z* 241.0833, 442.1617, and 744.2493, indicating the presence of the 6-methyl-octanoyl-Dab-Thr-Dab-Dab-Dab-Phe part of the PMB, which was similar to the results found in a different study [27] (Figure 3).

### 2.5. Tested Bacterial Strains Varied in Their Susceptibility to PMB

We assessed the effectiveness of PMB using several Gram-negative bacterial pathogens: three *Pseudomonas aeruginosa* strains (MRSN-17848, MRSN-18560, and MRSN-2108); one *Acinetobacter baumannii* strain (AB-10); and one *Klebsiellas pneumoniae* strain (Kp-UTI-2). The three P. aeruginosa strains are multidrug-resistant (MDR) isolates that were obtained from the Multidrug-resistant Organism Repository and Surveillance Network (MRSN) [28]. AB-10 was originally isolated from a patient with an infected wound, while KP-UTI-2 was obtained from a patient that presented to the Texas Tech University Health Sciences Center clinics with a urinary tract infection [29,30]. We first determined the minimum inhibitory concentration (MIC) using the reference broth microdilution method, as described by the Clinical and Laboratory Standards Institute (CLSI) [31]. As shown in Table 3 and according to the revised guidelines of the CLSI, all three *P. aeruginosa* isolates were resistant to PMB (interpreted breakpoint [BP] of ≥4 mg/mL). However, AB-10 was intermediate (interpreted BP of ≤2 mg/mL and KP-UTI-2 was susceptible (interpreted BP of ≤2 mg/mL (Table 3). According to the FDA-approved CLSI rationale, no susceptible BP is proposed for the revised BPs for polymyxin B regarding both *P. aeruginosa* and *A. baumannii*. Rather, the intermediate and resistant BPs were revised to ≤2 mg/mL and ≥4 mg/mL, respectively [32].

**Figure 4:**
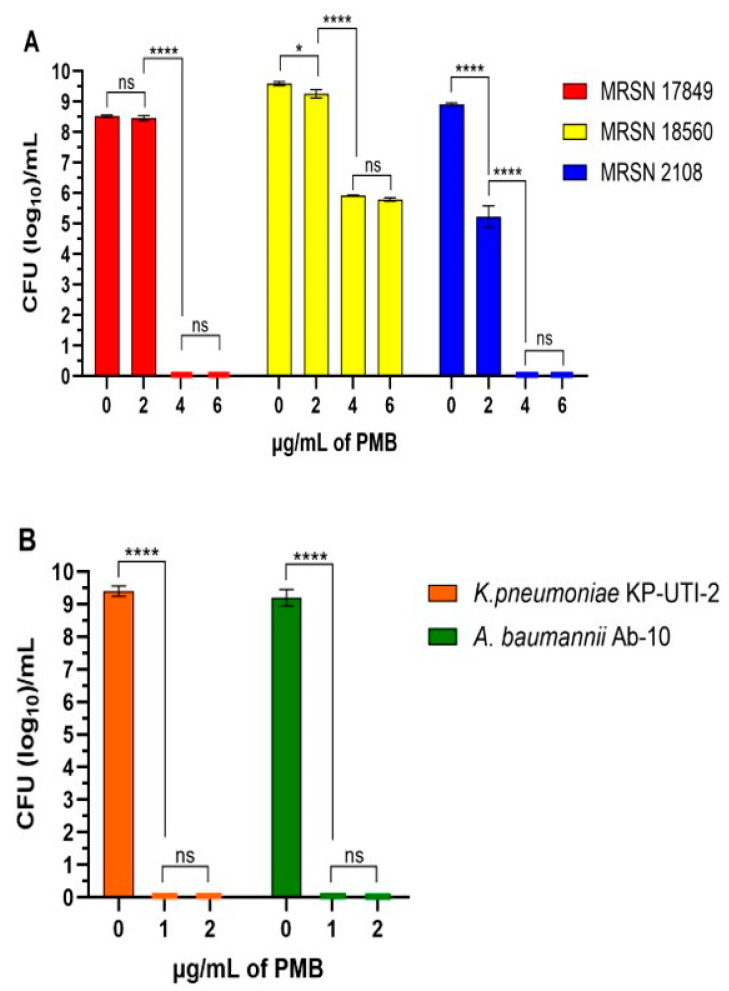
PMB inhibits the growth of several bacterial pathogens. The MBIC of PMB to each strain was determined as described in the Materials and Methods section. (**A**) The effect of PMB on three *P. aeruginosa* multidrug-resistant strains (MRSN 17849, MRSN 18560, and MRSN 2108. (**B**) The effect of PMB on the *K. pneumoniae* strain KP-UTI-2 and the *A. baumannii* strain AB-10. Bars indicate the means of three independent experiments. *, *p* < 0.05; ****, *p* < 0.0001; ns, not significant. Statistical significance (****) was determined using a two-way ANOVA with Tukey’s multiple comparison test. The growth of *P. aeruginosa* strains MRSN-17849 and MRSN-2108 was inhibited by 4 mg/mL (no CFU was recovered). Similarly, the *K. pneumoniae* strain KP-UTI-2 and the *A. baumannii* strain AB-10 were inhibited by 2 mg/mL. In the graphs, we included 4–5 CFUs for each point to conduct the statistical analysis.

Polymyxin B is bactericidal to different Gram-negative bacterial pathogens [32]. Therefore, besides determining the MIC, we assessed the minimal bactericidal concentration (MIC) of PMB to each pathogen using serial dilutions of 8, 4, 2, and 1 mg/mL. As shown in Figure 4A, the MBIC of PMB to both MRSN-17849 and MRSN-2108 is 4 mg/mL. However, while 2 mg/mL of PMB did not affect the growth of MRSN-17849, it reduced the growth of MRSN-2108 by 4 log_10_ (Figure 4A). In contrast, we could not determine the PMB MBIC to MRSN-18560. At 4, 6, 12, and even 20 mg/mL, the growth was only reduced by 3 log10 (Figure 4A). In contrast to *P. aeruginosa*, the MBIC of PMB to both AB-10 and PK-UTI-2 was 1 mg/mL (Figure 4B). Collectively, these results suggest that the tested pathogens varied in their susceptibility to PMB.

## 3. Discussion

All three tested *P. aeruginosa* isolates are MDR. Accordingly, and based on the results of the MIC assay, they were resistant at the breakpoint of ≥4 mg/mL (Table 3). However, based on the quantitative analysis (MBIC, CFU/mL), different concentrations of PMB produced three effect patters. MRSN-17849 was inhibited by 4 mg/mL, but no effect was detected at 2 mg/mL. MRSN-2108 was inhibit by 4 mg/mL, but its growth was significantly reduced by 2 mg/mL. MRSN-18560 was not inhibited by 4 mg/mL, but its growth was significantly reduced (Figure 4). It is interesting to note that beyond 4 mg/mL, the effect of PMB on the growth of MRSN-18560 was not concentration-dependent: no further reduction was seen regarding its growth, even at 30 mg/mL. In contrast to *P. aeruginosa*, the growth of either AB-10 or PK-UTI-2 was inhibited by 1 mg/mL, confirming their sensitivity (Figure 4). Polymyxin B is considered as one of the last defenses against important Gram-negative pathogens, such as *P. aeruginosa*, *A. baumannii*, and *Enterobacterales* [32]. However, with time and with the continuous use of the antibiotic polymyxin B, resistant mutants have emerged. The most important mechanism through which polymyxin B affects its target bacteria is through the association with the membrane and through the disruption of osmotic balance [33,34]. This occurs through the effect of the cation peptide and the hydrophobic fatty acid chain of the polymyxin B molecule. The cationic peptide region binds to the negatively charged lipopolysaccharide (LPS) of the bacterial outer membrane, whereas the fatty acid chain interacts with lipid A of the LPS. As a result, the membrane-stabilizing cations such as Ca^2+^ and Mg^2+^ are displaced [35,36]. Eventually, the outer membrane permeability barrier is compromised, causing the release of periplasmic proteins [37,38]. Other potential mechanisms of polymyxin B include the inhibition of bacterial respiration and the generation of reactive oxygen species [34,36,38]. Therefore, the primary mechanism of polymyxin B resistance is elicited through the modification of the charge on the outer membrane’s LPS moiety, thereby reducing the interaction with the polymyxin B molecule [39]. Thus, it is likely that although the three *P. aeruginosa* MRSN isolates are resistant to polymyxin B, they may vary in the degree of their LPS modification, which eventually influences the degree of polymyxin B resistance. Such a possibility may be tested by first screening more isolates through the MBIC assay and defining their level of resistance. Based on this, the LPS of selected isolates may be analyzed and compared.

A significant increase in antimicrobial resistance (AMR), particularly in those classified as ESKAPE pathogens (*Enterococcus faecium*, *Staphylococcus aureus*, *Klebsiella pneumoniae*, *Acinetobacter baumannii*, *Pseudomonas aeruginosa*, and *Enterobacter* species), poses a growing threat to global public health [40,41]. AMR infections contribute to 1.2 million deaths globally each year, and these numbers are projected to rise to 10 million by 2050. Of these deaths, Gram-negative bacterial pathogens pose a significant challenge, contributing to approximately 75% of the deaths attributed to antibiotic-resistant infections [42,43].

Polymyxin B, a member of the polymyxin family of antibiotics, is active against Gram-negative bacteria. However, conventional methods for identifying polymyxin B and its variants are time-consuming and inefficient. Leveraging bioinformatics to streamline antibiotic leads is a promising alternative. By employing genome-mining techniques, systematic profiling of the structural diversity of polymyxin-derived cationic peptides (CNRPs) can be carried out. This strategy allows for the identification and prioritization of promising polymyxin scaffolding, enabling further investigations.

## 4. Materials and Methods

### 4.1. Genome Mining

Genome sequences (11,218 in total, available on or before February 2019) were downloaded from the National Center for Biotechnology Information (NCBI) database. Sequences were subjected to non-ribosomal peptide screening using antiSMASH 4.0, including the presence of predicted cationic non-ribosomal peptides. Strains were further screened for commercial availability at the American Type Culture Collection (ATCC) and Leibniz Institute DSMZ.

### 4.2. Culture Conditions

Bacterial strains and growth media are listed in Supplementary Table S1. TSB-S20 cultures (1 L) were inoculated with 10 mL of 24 h culture (log phase) and grown for 48 h. (200 rpm, 30 °C) prior to centrifugation (10,000× *g*, 15 min). The cell-free supernatant was collected and assayed for biological activity.

### 4.3. Polymyxin Extraction

#### 4.3.1. Fermentation Broth Acid Precipitation

The supernatant was adjusted to pH 2.0 with HCl (100 mM) and incubated at 4 °C for 12 h; the precipitate was collected by centrifuging at 10,000× *g* for 15 min, and then it was re-dissolved in MeOH (100 mL). The solution was then centrifuged and concentrated in vacuo. The sample was re-suspended in ethyl acetate; after centrifugation (10,000× *g*, 10 min), the supernatant was dried and assayed for antibiotic activity.

#### 4.3.2. Resin Extractions

The final supernatant was bulk-absorbed onto selected resins, including Amberlite XAD-7, Diaion HP-20, and MCI gel CHP-20 at 10% (weight/vol). Further, it was incubated for 24 h at 4 °C. Resins were filtered (Whatman 226, 19.0 cm) and rinsed with deionized water (3 L) and 30% EtOH (1 L). Each resin was incubated with 70% EtOH (4 h) and adjusted to pH 2.0 (100 mM HCl). Resins were then filtered, and filtrates were concentrated in vacuo. Extracts were re-dissolved in water and assayed for activity.

### 4.4. In Vitro Antibiotic Assay

Initial screening of the antimicrobial effects was performed using previously described disk diffusion and the zone of inhibition (ZOI) assays derived from the Kirby-Bauer assay [44]. For the disk assay, overnight-grown strains were assayed by initially spreading them out uniformly on an LB-agar plate. A sterile filter paper disk (3 mm) impregnated with the test compound was placed on the surface of the plate. After a 24 h incubation period at 37 °C, the radius of bacterial inhibition was measured.

### 4.5. PMB Isolation and Purification

Amberlite XAD-7 samples (100 μL) were injected into an Agilent 1100 HPLC system equipped with a semi-preparative C18 column (300 Å, 5 µm, 10 mm × 250 mm) (WR Grace and Co., Vydac, Columbia, MD, USA). The mobile phase consisted of solvent A (HPLC water containing 0.1% trifluoroacetic acid (TFA)) and solvent B (acetonitrile with 0.1% TFA). A biphasic elution gradient consisted of B increasing from 18 to 35% over 24 min; increasing from 35 to 95% from 24 min to 43 min; and decreasing from 95 to 18% from 43 min to 50 min at a flow rate of 2.5 mL/min. The elution was monitored using a diode array detector (DAD) at 210, 214, and 280 nm. Fractions were collected every 5 min and concentrated in vacuo at 40 °C. The dried fractions were dissolved in sterile HPLC grade water and injected (30 μL) onto an analytical C18 column (300 Å, 5 µm, 2.1 mm × 250 mm) (WR Grace and Co., Vydac). The linear biphasic gradient increased from 18% to 40% B over 50 min. A single biologically active peak was isolated and concentrated in vacuo at 40 °C.

### 4.6. MALDI TOF MS Analysis

Purified PMB (2 μL, 1 mg/mL in MeOH) was combined with a 2 μL matrix solution containing 10 mg α-cyano-4-hydroxycinnamic acid dissolved in 1 mL of aqueous acetonitrile (50%, HPLC grade, with 0.1% TFA). The sample (1 μL) was spotted on a stainless steel plate and allowed to air-dry before being loaded into the MALDI (ABI SCIEX 4800 (MALDI-TOF/TOF)). Data were collected in positive ionization mode with an MS range of 400–2000 *m/z*; acquisition was carried out using a fixed laser intensity (4750 W/cm^2^) with the total shots per spectrum being 2500.

### 4.7. Minimum Inhibitory Concentration

Minimum inhibitory concentration (MIC) assay: the isolates were tested for susceptibility using the reference broth microdilution method, as described by the Clinical and Laboratory Standards Institute (CLSI) [31].

### 4.8. Assessing the Minimum Bactericidal Inhibitory Concentration (MBIC)

To determine the minimal inhibitory concentration (MBIC), plates with bacteria were incubated overnight on LB plates and diluted in a fresh LB to an initial inoculum of 10^6^ colony-forming units (CFUs)/mL and distributed in the wells (1 mL/well) of a 24-microtitre plate. The wells were divided into several sets (5/set) that were either untreated (control) or treated with variable concentrations (2, 4, 6 µg/mL) of the test compound. After a 24 h incubation period at 37 °C, 10-fold dilutions were prepared and assayed on inoculated LB-agar plates. The plates were incubated at 37 °C for 24 h, and the number of colonies produced by each dilution was counted. The total number of bacteria (CFUs/mL) was calculated using the following formula: CFUs counted × dilution factor × 100.

## Figures and Tables

**Table 1:** Predicted metabolites along with their net charges.

Organism	Metabolite
*Paenibacillus polymyxa*	(leu-thr-dab-dab) + (thr) + (dab-thr-dab-dab-dab)
	(ala) + (ala) + (thr-val-ala-thr-asn-ala)
	(mal) + (pk-tyr-ala) + (ala) + (ser) + (ser-ile-ser)
	(asn) + (ala) + (ala) + (ala-gly) + (mal) + (ala-ala) + (val)
	(val-ala-gly dab-trp-dab-ala-ala-trp-glu) + (val-ile) + (ile)
	(pk-ala) + (thr-ser-orn-ala-ala) + (phe-ala-ala)
	(leu-thr-dab-dab) + (thr) + (dab-thr-dab-dab-dab)
	(val-ala-gly dab-trp-dab-ala-ala-trp-glu) + (val-ile) + (ile)
	(ala) + (ala) + (thr-val-ala-thr-asn-ala)
	(mal) + (pk-tyr-ala) + (ala) + (ser) + (ser-ile-ser)
	(asn) + (ala) + (ala) + (ala-gly) + (mal) + (ala-ala) + (val)
	(pk-ala) + (thr-ser-orn-ala-ala) + (phe-ala-ala)
	(leu-thr-dab-dab) + (thr) + (dab-thr-dab-dab-dab)
	(pk-gly) + (pk) + (mal)

**Table 2:** Antimicrobial purification.

Step/Fraction	Sample Weight (mg)	Total Activity (AU/mg)	Specific Activity (AU/mg)
**Media Optimization**			
M 178	104.8	0	0
TSB	110.4	74.8	0.68
TSB-S20	183	172.7	0.94
TSB-S40	196.4	285.7	1.4
LB	175.2	35.3	0.20
kYPD	127.2	22.0	0.17
**Method of extraction**			
Acid precipitation	71.3	0	0
Amberlite XAD-7HP	573	502.4	0.88
MCI gel-HP	418.5	50.2	0.12
Diaion HP-20	162.8	22.0	0.13
**Chromatographic purification**			
HPLC timed fractionation	35.4	335.1	9.5
HPLC single-peak isolation	23.54	362.8	15.4

**Table 3:** Polymyxin B interpretative breakpoints according to the Clinical Laboratory Standards Institute (CLSI), 2020.

Strain	MIC (mg/mL)	Interpretation
*P. aeruginosa* MRSN 17849	≥4	Resistant
*P. aeruginosa* MRSN 18560	≥4	Resistant
*P. aeruginosa* MRSN 2108	≥4	Resistant
*A. baumannii* AB-10	≤2	Intermediate
*K. pneumoniae* KP-UTI-2	≤2	Susceptible

## Data Availability

Data is contained within the article or Supplementary Material, further inquiries can be directed to the corresponding author/s.

## Supplementary Materials

The following supporting information can be downloaded at https://www.mdpi.com/article/10.3390/antibiotics13080745/s1. Polymyxin supplementary data.
