# Supplementary material for: Nosocomial Bacteria Inhibition with Polymyxin B: In Silico Gene Mining and In Vitro Analysis
Source: Antibiotics (Basel). 2024 Aug 8;13(8):745. doi: 10.3390/antibiotics13080745 (PMC11350920; doi:10.3390/antibiotics13080745)
Supplement: Supplementary file 1 [file antibiotics-13-00745-s001.zip › antibiotics-3125396-supplementary.pdf]

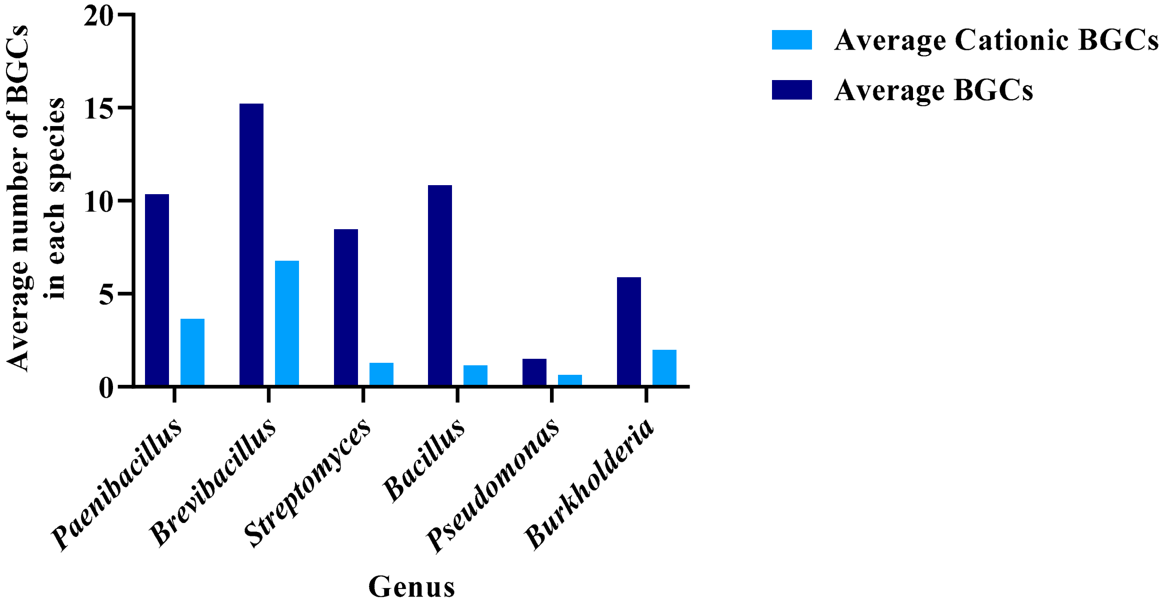

Figure S1. Distribution of biosynthetic gene clusters (BGCs) prediction hits by antibiotics and secondary metabolite analysis shell (antiSMASH). (Average number of BGC and cationic BGC present in each species).

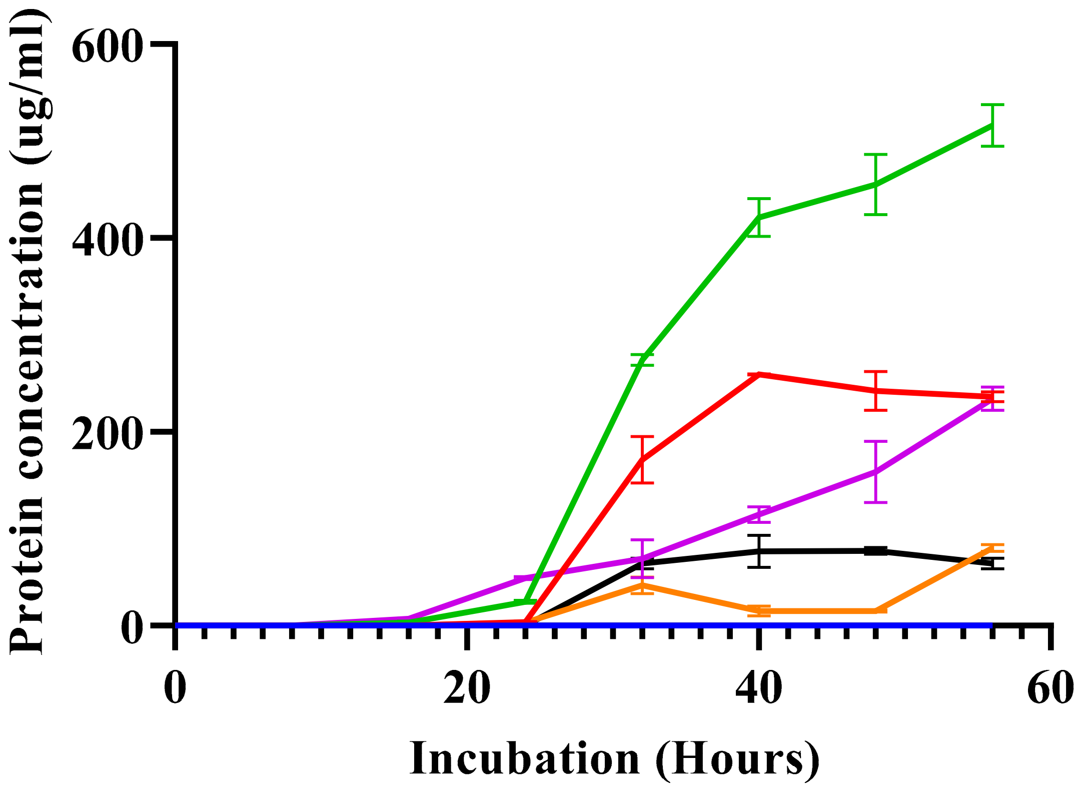

Figure S2. Supernatant protein amount from each medium. ATCC recommended media (M178, blue), tryptic soy broth (TSB, red), tryptic soy broth with starch (20g/L) (TSB S20, green), tryptic soy broth with starch (40g/L) (TSB S40, purple), Luria-Bertani broth (LB, orange) and yeast extract peptone dextrose (YPD, dark green)

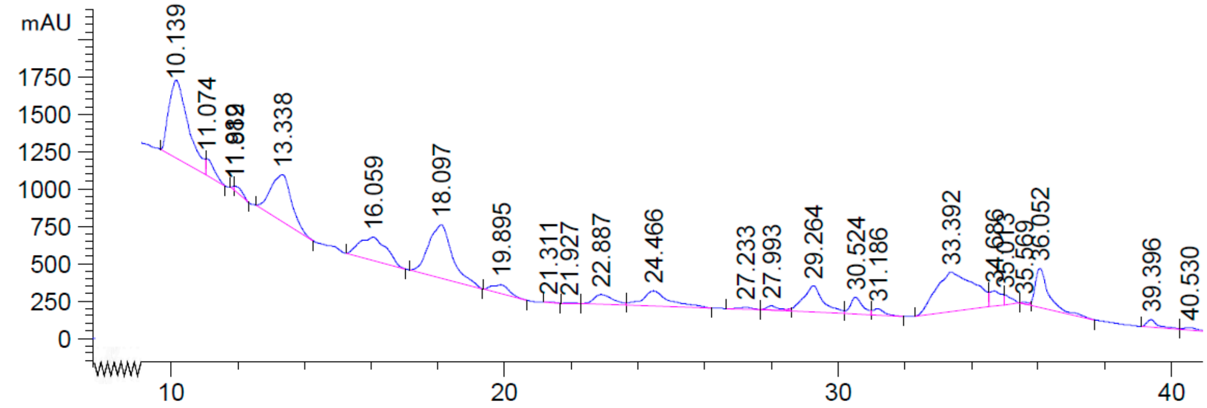

Figure S3. HPLC spectra of timed fraction collections of amberlite XAD-7 extract.

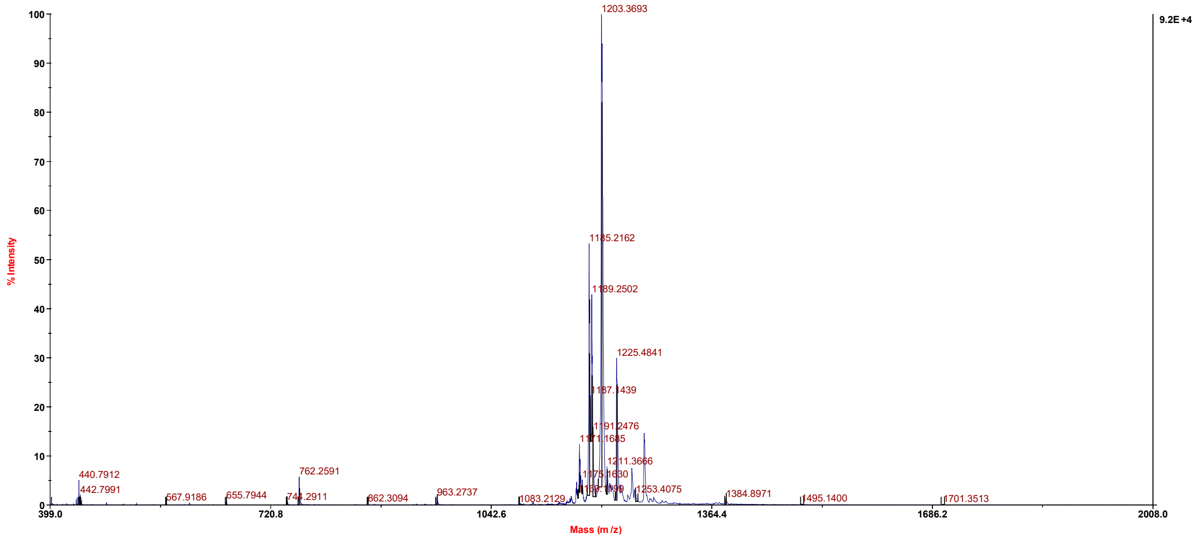

Figure S4. MALDI-TOF compound peak of isolated polymyxin B

Table S1. Strain characterization.

Strain	Description	Source/reference
Paenibacillus polymyxa ATCC15970	Nitrogen fixation bacteria isolated from soil.	American Type Culture Collection (ATCC)
Escherichia coli ATCC 9637	Susceptibility testing	ATCC
Pseudomonas aeruginosa MRSN 2108	The strain, which was originally isolated in 2011 from a human tissue, is part of the *P. aeruginosa* Diversity Panel provided by the Multidrug-resistant organism Repository and Surveillance Network (MRSN) at the Walter Reed Army Institute of Research (WRAIR)	The Biodefense and Emerging Infections Research Resources Repository (BEI Resources), NIH
Pseudomonas aeruginosa MRSN 17847	The strain, which was originally isolated in 2010 from a human wound, is part of the *P. aeruginosa* Diversity Panel provided by the Multidrug-resistant organism Repository and Surveillance Network (MRSN) at the Walter Reed Army Institute of Research (WRAIR)	BEI Resources, NIH
Pseudomonas aeruginosa MRSN 18560	The strain, which was originally isolated in 2013 from a human wound, is part of the *P. aeruginosa* Diversity Panel provided by the Multidrug-resistant organism Repository and Surveillance Network (MRSN) at the Walter Reed Army Institute of Research (WRAIR)	BEI Resources, NIH
Klebsiella pneumoniae UI-002, KP	*Klebsiella pneumoniae* isolate obtained from a patient with Urinary tract infection	Gynecology clinics, Texas The University Health Sciences Center, Lubbock, TX
Acinetobacter baumannii A118, ATCC-BAA-2093	The strain is a bloodstream isolate recovered in 1984 from a patient in an intensive care unit	ATCC
